# Pharmacological Treatment of Vascular Dementia: A Molecular Mechanism Perspective

**DOI:** 10.14336/AD.2020.0427

**Published:** 2021-02-01

**Authors:** Huang Kuang, Zhi-Feng Zhou, Yu-Ge Zhu, Zhi-Kai Wan, Mei-Wen Yang, Fen-Fang Hong, Shu-Long Yang

**Affiliations:** ^1^Department of Physiology, College of Medicine, Nanchang University, Nanchang, China.; ^2^Department of Nurse, Nanchang University Hospital, Nanchang 330006, Jiangxi, China.; ^3^Department of Experimental Teaching Center, Nanchang University, Nanchang, China.

**Keywords:** Vascular dementia, pharmacological treatment, oxidative stress, central cholinergic system, neuroinflammation, neuronal apoptosis, synaptic plasticity

## Abstract

Vascular dementia (VaD) is a neurodegenerative disease, with cognitive dysfunction attributable to cerebrovascular factors. At present, it is the second most frequently occurring type of dementia in older adults (after Alzheimer’s disease). The underlying etiology of VaD has not been completely elucidated, which limits its management. Currently, there are no approved standard treatments for VaD. The drugs used in VaD are only suitable for symptomatic treatment and cannot prevent or reduce the occurrence and progression of VaD. This review summarizes the current status of pharmacological treatment for VaD, from the perspective of the molecular mechanisms specified in various pathogenic hypotheses, including oxidative stress, the central cholinergic system, neuroinflammation, neuronal apoptosis, and synaptic plasticity. As VaD is a chronic cerebrovascular disease with multifactorial etiology, combined therapy, targeting multiple pathophysiological factors, may be the future trend in VaD.

Vascular dementia (VaD), characterized by progressive cognitive dysfunction, is generally considered the second most common type of dementia in older adults, after Alzheimer's disease (AD). VaD accounts for approximately 15% to 20% of dementia cases in North America and Europe [1], and has somewhat higher estimates, around 30%, in Asia and developing countries [2]. In China, the incidence of VaD is 2.42 per 1000 person-years in individuals aged 60 years or above [3, 4]. Although patients with VaD constitute the second largest dementia population in the world, treatment data are lacking. VaD causes a continuous and irreversible deterioration in the quality of life, resulting in huge medical and economic burdens on families and society.

VaD has multifactorial etiopathology, diverse clinical manifestations, and multiple clinical subtypes. Moreover, the diagnostic criteria are not consistent worldwide. Current international diagnostic criteria mainly include the DSM-5 [5], ICD-11 [6], VASCOG [7], ADDTC [8], and NINDS-AIREN [9]. Although these clinical diagnostic criteria have varying features, they all contain the following three elements: (1) diagnostic criteria for dementia are met; (2) there is evidence of cerebrovascular disease; and (3) there is a mutually causal relationship between dementia and cerebrovascular disease [10]. Unfortunately, ideal indicators for clinical diagnosing are lacking. Pathological examination is the only gold standard for diagnosis, but its implementation is difficult. The Hachinski Ischemic Scale (HIS) can also be used for the identification of VaD and AD [11]. Because of its convenient implementation and high reliability, the HIS is widely used in the clinic. In recent years, the concept of vascular cognitive impairment (VCI) was delineated, with classifications of VCI-no dementia, VaD, and mixed dementia (AD and VaD) [12]. The introduction of VCI has mitigated shortcomings in the clinical diagnosing of VaD, greatly advancing the diagnostic time and providing a basis for early intervention.

However, the pathogenesis of VaD still remains unclear. Although the symptoms of VaD are similar to those in AD, patients with VaD show a chronic decrease in cerebral blood flow, which differs from the brain changes in AD [13]. Several types of vascular lesions can trigger the molecular mechanisms leading to VaD, including chronic cerebral hypoperfusion (CCH), small vessel disease (leukoaraiosis and lacunar infarcts), microinfarcts, microhemorrhages, cerebral amyloid angiopathy, and mixed vascular lesions [14, 15]. CCH has been reported as the main cause of VaD [14, 16]. CCH can lead to a series of pathophysiological changes that contribute to cognitive deficits, eventually causing VaD. CCH-induced VaD animal models have implicated oxidative stress, central cholinergic system dysfunction, neuroinflammation, neuronal apoptosis, and synaptic plasticity dysfunction in the pathogenesis of VaD. Thus, most of the drug targets in VaD are based on these five pathophysiological changes.

Currently, there are no approved standard treatments for VaD. Furthermore, the drugs used in VaD are only suitable for symptomatic treatment and cannot prevent or delay the course of VaD [17]. Therefore, novel treatments in VaD are urgently needed. Based on the known pathophysiological changes in VaD, a variety of pharmacological treatment approaches have been proposed. The objective of this report is to review the current progress in research regarding relevant therapeutic strategies and mechanisms in VaD.

## 1. Inhibition of oxidative stress

Brain oxidative stress resulting from CCH is recognized as a major factor among the potential mechanisms of VaD. Moreover, increased oxidative stress injury is considered to be a critical mechanism underlying cognitive deficits in VaD [14]. When brain ischemia and ischemia-reperfusion injuries occur, a large amount of lipid peroxidation products and free radicals are generated during oxidative stress. At this point, changes in cell membrane phospholipids lead to increased permeability, which promotes excessive edema and the release of excitatory transmitters. Finally, a series of chain reactions cause the demise of neurons [18]. The amount of neuronal necrosis continues to grow, and the size of the infarction increases, eventually leading to VaD. Hence, drugs that inhibit oxidative stress show promise as a treatment for VaD.

### 1.1 Reduction of lipid peroxidation products

Malondialdehyde (MDA) and 4-hydroxynonenal (4-HNE) are common accessory substances of lipid peroxidation generated under oxidative stress conditions and are recognized as evaluation indexes for the degree of oxidative damage. An intermediate product of lipid peroxidation, reactive oxygen species (ROS), and the final decomposition products, MDA and 4-HNE, cause severe damage to the membrane structure and function, ultimately resulting in cytotoxicity [19]. Thus, reducing the production of intracellular lipid peroxidation products can have an antioxidant effect.

Tetramethylpyrazine nitrone (TBN) has shown neuroprotective properties in VaD rats through its antioxidant ability [20]. Studies have shown that TBN significantly suppresses the 4-HNE production in white matter and rescues cognitive deficits in VaD rats [20]. Alpha-lipoic acid (ALA) is a naturally occurring disulfide. It is considered to be a suitable neuroprotective agent, as it can cross the blood-brain barrier and be absorbed evenly in all parts of the central nervous system (CNS) [21]. Studies have revealed that ALA administration markedly reduces MDA and ROS production in the hippocampus of CCH-induced VaD rats and improves learning and memory abilities. Therefore, it has been suggested that ALA improves cognitive deficits partly via its antioxidant effects [22]. Additionally, betain [23] and betulinic acid (BA) [24], two natural drugs, exert neuroprotective effects, similar to those of ALA, by suppressing oxidative stress in VaD rats. Furthermore, estrogen deficiency leads to increased free radical and lipid peroxidation production and decreased antioxidant enzymatic activity, resulting in reduced free radical-scavenging activity [25, 26]. Biochanin A (BCA, a phytoestrogen) is an estrogen receptor ligand similar to estrogen. BCA effectively reduces oxidative damage by diminishing lipid peroxidation production, thus markedly improving learning and memory functions in VaD rats [27].

### 1.2 Promotion of free radical scavenging

As free radicals are thought to cause cognitive dysfunction, it is sensible to seek compounds that have antioxidant and neuroprotective properties against VaD. The activities of antioxidant enzymes, such as superoxide dismutase (SOD), catalase (CAT), and glutathione (GSH), are decreased in patients with VaD [28, 29]. Furthermore, hypertension has been identified as a risk factor for VaD [30]. Studies have shown that hypertension is closely linked to the long-term risk of dementia and cognitive dysfunction [31, 32].

Antihypertensive treatment can reduce the risk of cognitive impairment by preventing the incidence of cerebrovascular diseases, such as VaD. In animal models with hypertension-induced VaD, the administration of TWK10-fermented soymilk extract improves cognitive function by inhibiting oxidative stress [33]. The administration of TWK10-fermented soymilk extract also increases the scavenging activity of *α,α*-diphenyl-*β-*picrylhydrazyl (DPPH) and the activity levels of CAT, GSH, and SOD [33]. Additionally, 1-phenylisatin, a selective cannabinoid receptor type 2 (CB2R) agonist, has the same effects as those of TWK10-fermented soymilk extract in CCH-induced VaD rats [34]. Edaravone is a novel free radical scavenger that ameliorates neuronal damage and exerts neuroprotective effects in neurodegenerative diseases [35, 36]. Edaravone markedly inhibits oxidative stress by elevating SOD activity and reducing MDA and ROS levels in the hippocampus of VaD rats. Additionally, the administration of edaravone rescues the cognitive deficits in VaD rats [37]. Furthermore, simvastatin exerts antioxidant properties by scavenging ROS, instead of by lowing blood lipids [38]. Simvastatin also increases GSH activity and reduces MDA levels in the brain of animals with L-methionine-induced VaD, ameliorating L-methionine-induced cognitive decline to some extent [39]. In addition, melatonin has neuroprotective properties against CCH injury, attributable to its antioxidant ability [40]. Melatonin increases SOD, CAT, and GSH levels, as well as the total antioxidant capacity (TAC), by upregulating senescence marker protein-30 (SMP30) and osteopontin (OPN) in the hippocampus of VaD rats [41]. Thus, free-radical scavengers, such as melatonin, may be a treatment option to suppress oxidative stress and improve cognitive function in patients with VaD.

Natural drugs with active ingredients also hold great promise in the treatment of VaD. Administrations of the Sailuotong (SLT) formula [42], Jiji decoction [43], alpha-naphthoflavone (ANF) [44], and *in-vitro* cultured calculus bovis (ICCB) [45], inhibit oxidative stress by increasing SOD and GSH activity in the hippocampus of VaD rats, markedly rescueing the cognitive deficits. SLT, a three-herb formula composed of *Panax ginseng*, *Ginkgo biloba*, and *Crocus sativus*, has been used to treat VaD [46]. Moreover, two random clinical trials (RCTs) have explored the efficacy and safety of SLT in VaD [47, 48]. In one clinical trial, 227 patients were randomly divided into experimental groups (SLT 360 mg [114 patients] or 240 mg [113 patients] for 52 weeks) or control groups (SLT 360 mg or 240 mg [113 patients each] only from weeks 27 to 52). After treatment, the experimental groups had improved scores compared to those of the control groups [48]. Jiji decoction consists of six medicinal plants: *Coptis chinensis, Alpinia oxyphylla Miq., Wolfberry, Cuscuta, Radix Rehmanniae*, and *Scutellaria baicalensis*. In addition to improving the cognitive dysfunction in VaD rats, Jiji decoction significantly improves learning and memory functioning in normal animals [43]. The above results show the great advantages of natural drugs in VaD treatment, which may indicate a future research direction in VaD therapy. However, more relevant RCTs are needed to explore the safety and efficacy of these drugs.

### 1.3 Regulation of relevant oxidative stress pathways

The nuclear factor erythroid 2-related factor 2 (Nrf2)/heme oxygenase-1 (HO-1) signaling pathway is an important antioxidant stress signaling pathway. When activated, it regulates the release of antioxidant substances, thereby inhibiting oxidative stress [49]. Nrf2 is a primary transcription factor involved in regulating the body's natural antioxidative stress defense system. When stimulated by oxidative stress, it initiates the expression of the downstream gene, HO-1, and plays a protective role in antioxidative stress [50]. Under physiological conditions, Nrf2 binds to Kelch-like ECH-associated protein 1 (Keap1). Under oxidative stress, Nrf2 is released from the Keap1-Nrf2 complex and transferred from the cytoplasm to the nucleus, where it binds to antioxidant response elements located in HO-1 promoter regions [51, 52].

**Figure 1.**
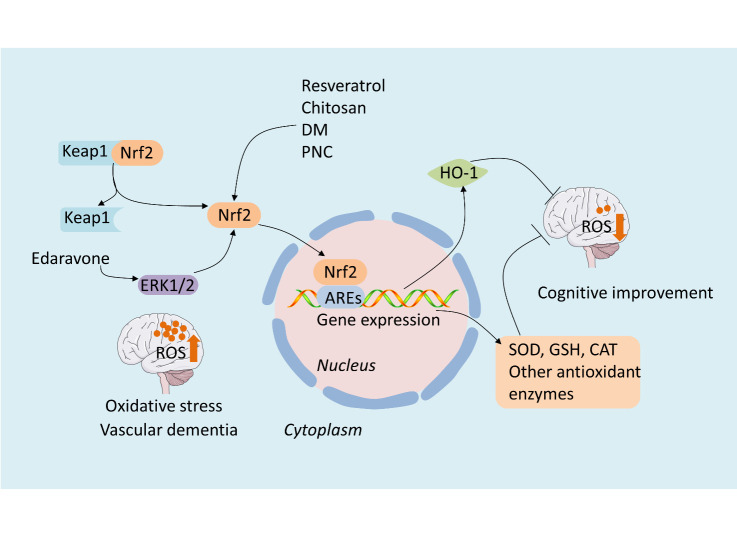
Drugs treat VaD through regulating the Nrf2/HO-1 signaling pathway. The Nrf2/HO-1 pathway plays an essential role in inhibiting the oxidative stress in the targets of treatment of VaD. In the case of oxidative stress, Nrf2 is released from the Keap1-Nrf2 complex, transferred from the cytoplasm to the nucleus, and binds to the AREs, promoting the expression of HO-1 and other antioxidant enzymes such as SOD, GSH, and CAT. These antioxidant substances could promote free radical scavenging and exert antioxidant and neuroprotective effects on VaD. Resveratrol, chitosan, DM and PNC could up-regulate the expression of Nrf2, initiating the Nrf2/HO-1 pathway, thus inhibiting the oxidative damage and alleviating the cognitive deficits of VaD. ERK phosphorylation is the core pathway implicated in the activation of Nrf2. Edaravone could enhance the expression of ERK1/2 and then activate the Nrf2/HO-1 pathway, exerting neuroprotective effects on VaD.

Edaravone [53], resveratrol [54], chotosan [55], and dextromethorphan [56] can inhibit oxidative stress, improve the antioxidant capability in VaD model animals, exert neuroprotective effects, and alleviate cognitive impairment via the Nrf2/HO-1 pathway (Fig. 1). More specifically, edaravone increases SOD activity, while decreasing MDA levels in the hippocampus via Nrf2 signaling pathway activation, which is associated with extracellular regulated protein kinases (ERK1/2) [53]. It is well established that ERK phosphorylation is the core pathway implicated in the activation of Nrf2 [57]. Chotosan not only promotes HO-1 expression through the Nrf2 pathway, but also enhances the expression of NADPH/quinone oxidoreductase 1 (NQO1), and exerts better neuroprotective effects compared to those of edaravone [55]. Additionally, the co-administration of aripiprazole and cilostazol collectively suppressed oxidative stress-induced neurotoxicity through the ERK/Nrf2/HO-1 pathway in H_2_O_2_-induced HT22 cells [58]. This provides a theoretical basis for the use of combination pharmacological treatment in VaD.

NADPH oxidase (Nox), an enzyme complex that transfers electrons from the membrane and produces ROS, is involved in pathological conditions in neuronal cells [59]. It is well known that flavanones have free radical-scavenging activity [60]. (*2R,3S*)-pinobanksin-3-cinnamate (PNC) is a new type of flavonoid extracted from *Alpinia galanga Willd.*, with a neuroprotective effect on H_2_O_2_-damaged cells. Recent studies have demonstrated that PNC markedly reduces ROS levels, while elevating SOD and GSH activity, in VaD rats, thereby improving behavioral performance [61]. Specifically, these data suggest that PNC elevates Nrf2 levels, while reducing Nox1 levels, as the potential mechanism underlying its effects [61]. Therefore, balanced regulation between the Nox-dependent oxidation pathway and the Nrf2-dependent antioxidant pathway might provide benefits in the management of VaD.

## 2. Restoration of the central cholinergic system

The central cholinergic system dysfunction has been found in AD cases, and acetylcholinesterase (AChE) inhibitors are presently the most commonly used agents in the treatment of AD [62, 63]. Similarly, cholinergic dysfunction has also been documented in VaD cases [64]. Therefore, patients with VaD may also benefit from central cholinergic system restoration. Current evidence suggests that cholinergic drugs may be utilized for symptomatic treatment of patients with VaD with cognitive impairment [65].

### 2.1 Inhibition of AchE activity

The presence of significantly elevated AChE activity indicates cholinergic damage in the hippocampus, which triggers cognitive deficits, in patients with VaD [64]. As in AD, AChE inhibitors may exert benefits in patients with VaD. This is the theoretical basis for the early use of AChE inhibitors to alleviate VaD symptoms.

Although there are currently no officially approved drugs for VaD treatment, AChE inhibitors in VaD are widely used in clinical practice. Evidence from numerous studies, including RCTs, supports the advantages of AChE inhibitor drugs, such as donepezil [66-70], galantamine [71-74], and rivastigmine [75-78]. The administration of these agents results in significant improvements in cognition, global function, and ability. However, AChE inhibitors do not always provide consistent cognitive results among potential cases of VaD in elderly patients. This suggests that the putative cholinergic deficiency in VaD may reflect the presence of concomitant AD pathology. Therefore, it is necessary to establish specific clinical diagnostic criteria for VaD and perform multi-center, large sample, long-term RCTs to better evaluate the efficacy of AChE inhibitor drugs in VaD.

In addition to the three AChE inhibitors mentioned above, other AChE inhibitors may have protective effects against VaD. Administration of the estrogen analogue, BCA, markedly reduces AChE activity in VaD rats [27]. Ryanodine and cysteinyl leukotriene 1 receptors (RyRs and CysLT1Rs, respectively) are widely present in the CNS and are implicated in cognition and inflammation regulation [79]. Administration of ruthenium red (a RyRs antagonist) and montelukast (a CysLT1Rs antagonist) considerably attenuates CCH-induced cholinergic dysfunction by suppressing AChE activity, reducing the cognitive impairment and brain damage [80]. Thus, the modulation of RyRs and CysLT1Rs may provide a therapeutic target in CCH-induced disorders, such as VaD.

Hwangryunhaedok-tang (HRT) is a four-herb formulation consisting of the following medicinal plants in a 1:1:1:1 ratio: *Phellodendri Cortex*, *Scutellariae Radix*, *Coptidis Rhizoma*, and *Gardeniae Fructus* [81]. HRT inhibits AChE activity, restores acetylcholine (Ach) levels, and alleviates memory impairments and neuronal damage in CCH-induced VaD rats [64]. These results demonstrate the therapeutic effects of HRT as a cholinergic drug for VaD. Additionally, simvastatin [39], ST09 (a novel thioester derivative of 6-chloro-tacrine) [82], and the co-administration of melatonin and resveratrol [83] significantly decrease AChE activity and rescue cognitive deficits in VaD rats. Although these results are encouraging, RCTs are still needed to evaluate the safety and efficacy of these agents in the treatment of VaD.

### 2.2 Promotion of ChAT expression

Ach is an important neurotransmitter involved in learning and memory functions of the CNS. Accordingly, decreased Ach synthesis inevitably leads to cognitive dysfunction and, eventually, VaD. Studies have reported that choline acetyltransferase (ChAT) activity, a synthetic bioenzyme for Ach, is reduced in the hippocampus of VaD rats [84, 85]. BCA [27] has estrogen-like effects and can enhance ChAT expression in rat hippocampal cholinergic neurons; thus, BCA exhibits a certain degree of protection against memory impairment in VaD rats. Additionally, some scholars have proposed that estrogen can improve VaD symptoms in model animals by increasing the calcium load and calbindin-d28k [86] and VEGF [87] expression, suggesting that estrogen plays an anti-VaD role via multiple molecular mechanisms. Erythropoietin (EPO) also has potential therapeutic use, in terms of neuroprotection, as studies have demonstrated that EPO can improve white integrity after cerebral ischemia [88, 89]. EPO administration rescues cholinergic dysfunction through the restoration of ChAT activity and Ach levels, and the inhibition of AchE activity, in VaD rats [90]. These data support the behavioral effects, and suggest that EPO may exert its neuroprotection by rescuing cholinergic function. In addition, ALA administration rescues cholinergic dysfunction by increasing ChAT activity and Ach levels [22]. Thus, the neuroprotective properties of ALA in the central cholinergic system represent another mechanism for treating VaD.

Additionally, natural drugs, such as the ethanolic extract of *Fructus mume* (*F. mume*) from the unripe fruit of *Prunus mume*, have been reported to reverse cognitive deficits caused by CCH via central cholinergic system restoration [91, 92]. Moreover, *F. mume* administration restores ChAT expression in the hippocampus, suggesting that *F. mume* is a promising agent for the treatment of VaD [93].

## 3. Inhibition of neuroinflammation

Neuroinflammation is an important process in the etiology of VaD. The memory recovery effect is associated with cerebral ischemia-induced expression of inflammatory factors [94]. The expression levels of various inflammatory cytokines in brain tissue are abnormally increased in VaD [95]. Additionally, increased inflammatory responses, such as the activation of main inflammatory cells in brain tissue [95] and inflammation-related signaling, have been reported in animal models of VaD [64]. Accordingly, drugs that only target neuronal protection cannot achieve the expected results. Therefore, proper consideration should be given to the role of neuroinflammatory responses in VaD, as this is conducive to the development of new drugs.

### 3.1 Suppression of inflammatory cytokines

The inflammatory response leads to an up-regulation of pro-inflammatory cytokines, such tumor necrosis factor-α (TNF-α), interleukin-6 (IL-6), and interleukin-1β (IL-1β), and can aggravate the degree of neuroinflammation after CCH [24].

The CB2R agonist, WIN55,212-2 (WIN), has neuroprotective effects in focal cerebral ischemia [96] and AD [97]. Recent studies have shown that IL-1β and TNF-α are markedly elevated in VaD rats, accompanied by increased autophagy. However, after the administration of WIN, the levels of inflammatory cytokines (IL-1β and TNF-α), and expression of autophagy-related factors [microtubule-associated protein 1 light chain 3 (LC-3) and beclin-1], were significantly suppressed. Additionally, behavioral experiments indicate that WIN rescues the cognitive deficits in VaD rats [98]. These results indicate that WIN exerts neuroprotective properties, supporting cognition function in VaD rats, potentially related to the inhibition of inflammation and abnormal autophagy. Similarly, another selective CB2R agonist, paeoniflorin (PF), can significantly attenuate CCH-induced impairments in learning and memory in VaD rats. In addition to inhibiting pro-inflammatory cytokines, such as IL-1β, IL-6, TNF-α, and NO, PF also significantly up-regulates the expression of anti-inflammatory cytokines, IL-10 and transforming growth factor beta 1 (TGF-β1) [99, 100]. Based on the above research, three selective CB2R agonists, 1-phenylisatin, WIN, and PF, can improve cognitive impairments in VaD via various molecular mechanisms, suggesting that they constitute a very promising target for pharmacological treatment in VaD.

Additionally, the administration of bee venom (BV) has been shown to alleviate cognitive impairment and decrease the expression levels of neuroinflammatory cytokines, including ionized calcium binding adaptor molecule 1 (Iba-1), CD14, and TNF-α, in the hippocampus of VaD rats [101]. Moreover, the underlying mechanism of the pharmacological effect of BV may be mediated via the neuroinflammation-related toll-like receptor 4 (TLR4) signaling pathway [101].

### 3.2 Suppression of glial activation

Chronic cerebral ischemia causes the proliferation and activation of glia, such as microglia and astrocytes, thereby producing a large number of inflammatory cytokines and oxygen free radicals, which are implicated in the pathogenesis of VaD [102, 103]. CD11b [104] and glial fibrillary acidic protein (GFAP) [105] are characteristic markers of microglia and astrocytes, respectively, and their expression reflects the proliferation and activation of both types of cells.

The administration of Yangxue Qingnao granules (YQG) can rescue the elevated expression of CD11b and GFAP in the CA1 region of the hippocampus in VaD rats [106]. YQG includes *Radix angelicae sinensis*, *Radix paeoniae alba*, *Rhizoma chuanxiong*, *Caulis spatholobi*, *Radix rehmanniae preparata*, *Ramulus uncariae cum uncis*, *Spica prunellae*, *Semen cassiae*, *Concha margaritifera usta*, *Rhizoma corydalis yanhusuo*, and *Herba asari* [107]. This result indicates that YQG may play a therapeutic role in VaD by inhibiting glial proliferation and activation. Additionally, carnosine administration suppresses glial activation by attenuating the elevated expression levels of GFAP and Iba-1, thereby ameliorating the white matter lesion and cognitive impairments [105]. Iba-1 is another marker of microglia activation and has significantly increased expression in VaD rats [101]. Scutellarin (SCT), a major flavonoid in the medicinal herb of *Erigeron breviscapus* (*vant.*) Hand. Mazz., also exhibits neuroprotective effects. SCT significantly inhibits the activation of Iba1-expressing microglia in brain tissues, and ameliorates cognitive impairments caused by CCH [108]. Palmitoylethanolamide (PEA) is a prototype ALIAmide, well-known for its analgesic, anti-inflammatory, and neuroprotective properties. Recent studies have demonstrated that PEA oxazoline (PEA-OXA) administration significantly alleviates the pathological changes and neuronal death caused by CCH and rescues behavioral dysfunction [109]. Furthermore, PEA-OXA reduces GFAP and Iba-1 expression, thereby inhibiting the activation of microglia and astrocytes [109]. Thus, PEA-OXA may become a new target for suppressing the neuroinflammatory response in VaD. Additionally, recent studies have reported that SLT administration suppresses neuroinflammation by inhibiting astrocytic reactions in VaD rats [94].

### 3.3 Regulation of inflammation-related signaling pathways

The role of inflammasomes in neurodegenerative diseases has been recently described [110, 111]. Furthermore, elevated hippocampal NLR family pyrin domain containing 3 (NLRP3) expression indicates that the up-regulation of NLRP3 is related to CCH-induced neuroinflammation [112, 113]. Notably, NLRP3 inflammasome activation initiates the proteolytic activity of caspase-1, which is necessary for the secretion of the inflammatory cytokine, IL-1β [114]. Additionally, nuclear factor kappa B (NF-κB) signaling is a well-known neuroinflammatory regulatory pathway. NF-κB is an important transcription factor in the inflammatory response. Cerebral ischemia and hypoxia can cause a large amount of NF-κB activation, promoting inflammatory infiltration and increasing the expression levels of inflammatory cytokines, such as TNF-α and IL-1β [115, 116]. Furthermore, the mitogen-activated protein kinase (MAPK) signaling pathway is involved in the process of microglial activation. Lipocalin-2 (LCN2) is another inflammatory factor that is a target in regulating astrocytic reactions, and plays a detrimental role in the ischemic brain and neurodegenerative diseases [117, 118].

3-[2-[4-(3-chloro-2-methylphenyl)-1-piperazinyl] ethyl]-5,6-dimethoxyindazole (DY-9836), a calmodulin inhibitor [119], can mitigate hippocampal-dependent cognitive deficits in VaD rats. Moreover, these neuroprotective properties are linked to a significant reduction in peroxynitrite formation, which is associated with down-regulation of NLRP3/caspase-1/IL-1β signaling [112]. Additionally, sirtuin 1 (SIRT1) modifies the Lys310 residue of RelA/P65 by deacetylation, and inhibits NF-κB transcriptional activity, thereby reducing its inflammatory response and the expression of inflammatory cytokines [120]. Triptolide administration improves cognitive impairments in VaD rats, increases SIRT1 expression, and decreases NF-κB expression; thus, its mechanism may be related to SIRT1/NF-κB signaling [121]. In addition, mammalian target of rapamycin complex 1 (mTORC1) is an essential factor in regulating glial functions. Studies have suggested that everolimus (RAD001), a rapamycin analogue, suppresses mTORC1 expression, and ameliorates cognitive deficits through microglia M1/M2 balance restoration in VaD rats [122]. Thus, RAD001 is considered as a potential pharmacological treatment for VaD.

**Figure 2.**
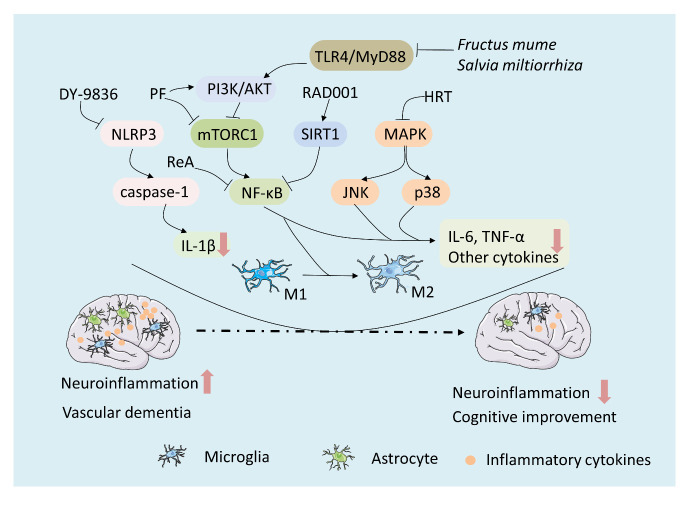
Drugs treat VaD through regulating inflammation-related signaling pathways. There are several inflammation-related pathways involved in neuroinflammation of VaD, which can be used as pharmacological targets. The NF-κB signaling is a core pathway among these inflammation-related pathways. Some drugs such as ReA, PF, *Fructus mume*, *Salvia miltiorrhiza*, and RAD001 can act directly on NF-κB or its upstream molecules, thereby inhibiting the pro-inflammatory pathways, reducing expression of inflammatory cytokines, suppressing the activation of glia and promoting the polarization of M1/M2. These effects are beneficial to the cognitive improvement of VaD. Other inflammation-related signaling pathways, such as NLRP3/caspase-1/IL-1β and MAPK signaling could be inhibited by DY-9836 and HRT respectively. Hence, these drugs can exert a neuroprotective effect on VaD.

In addition to alleviating cognitive dysfunction through cholinergic dysfunction rescue, the herbal formula, HRT, can inhibit neuroinflammation by regulating p-Jun N-terminal kinase (JNK) and p38 MAPK pathways in the hippocampus of VaD rats [64]. Additionally, other natural drugs, such as *F. mume* [123] and *Salvia miltiorrhiza* [124], can alleviate cognitive impairments through the inhibition of neuroinflammation by down-regulating TLR4/myeloid differentiation factor 88 (MyD88) and p38 MAPK signaling. Moreover, the CB2R agonist, PF [99], has been shown to exert an anti-neuroinflammatory effect in VaD rats, by significantly inhibiting CCH-induced mammalian target of mTORC1/ NF-κB pro-inflammatory pathway signaling and enhancing phosphatidylinositol-3 kinase (PI3K)/ protein kinase B (AKT/PKB) anti-inflammatory pathway signaling [99] (Fig. 2). Furthermore, treatment with SLT decreases LCN2 expression and the phosphorylation levels of Janus kinase-2 (JAK2) and signal transducer and activator of transcription-3 (STAT3) and mitigates the cognitive deficits in VaD rats. These results suggest that SLT inhibits neuroinflammation in VaD rats via the LCN2-JAK2/STAT3 pathway, providing insight into a promising therapeutic strategy in VaD [94]. Finally, rehmannioside A (ReA) is isolated from *Rehmanniae Radix* and exhibits a protective role against VaD. Studies have found that the administration of ReA attenuates the cognitive deficits and histological alterations of the hippocampus in VaD rats, partly due to the inactivation of NF-κB [125].

## 4.Inhibition of neuronal apoptosis

VaD is a multifactorial disorder. The mechanism of neuronal apoptosis is of great significance for understanding its pathogenesis. Emerging data demonstrate that cell survival regulation requires a series of proteins associated with apoptosis, including Bax, B-cell lymphoma-2 (Bcl-2), and caspase-3. However, their expression is abnormal in VaD. Inhibiting neuronal apoptosis may be beneficial in treating VaD [42, 122].

### 4.1 Suppression of caspase-3 activation

Caspase-3 is the main terminal cleaving enzyme in the process of apoptosis. Caspase-3 expression is elevated considerably in VaD rats [42, 126]. Administration of ReA [122], ligustilide [127], osthole [128], and SLT [42] has been reported to prevent caspase-3 activation and reverse the loss of hippocampal neurons, as well as the cognitive deficits, induced by CCH. In addition to inhibiting oxidative stress and AchE activity, the natural drug resveratrol (mentioned above), can also suppress neuronal apoptosis by reducing apoptotic proteins, such as caspase-3, in the hippocampus of VaD rats, thereby relieving the symptoms of VaD [129]. These results indicate that anti-apoptotic effects mediated by the suppression of caspase-3 activation might contribute to improvements in cognitive function in VaD.

### 4.2 Suppression of Bax/Bcl-2 ratio

The Bax/Bcl-2 ratio also plays a role in cell apoptosis [130, 131]. Bcl-2 and Bax are two major proteins that regulate apoptosis and have opposing functions. Bcl-2 is a functional inhibitor of apoptosis, while Bax is a promoter of apoptosis. A higher Bax/Bcl-2 ratio indicates stronger pro-apoptotic ability, and a lower ratio indicates less pro-apoptotic ability. Immunohistochemical results demonstrate that Bax expression is remarkably increased, and Bcl-2 expression is decreased, in VaD rats, resulting in a high Bax/Bcl-2 ratio [45, 126]. The administration of ICCB [45], osthole [128], PNC [61], Panax ginseng [132], total harmaline alkaloid extract [133], and SLT [42] reduces the Bax/Bcl-2 ratio and exerts anti-apoptotic properties in the brain of VaD rats, alleviating the cognitive deficits. Based on the above research, the Chinese medicinal formula, SLT, can improve VaD symptoms via multiple mechanisms, including oxidative stress, neuroinflammation, and neuronal apoptosis. These results indicate SLT as a promising drug for the treatment of VaD.

### 4.3 Regulation of apoptosis-related pathways

The inflammatory cytokine, monocyte chemotactic protein 1 (MCP-1), and oxidative stress factor, homocysteine (Hcy), are involved in promoting apoptosis by up-regulating the Bax/Bcl-2 ratio, while brain-derived neurotrophic factor (BDNF) acts as an anti-apoptotic factor by down-regulating MCP-1 and Hcy [126]. MCP-1 and Hcy expression levels are markedly elevated, while the BDNF level is significantly reduced, in VaD models. The PI3K/AKT signaling pathway can also down-regulate the Bax/Bcl-2 ratio, alleviating neuronal apoptosis [134]. Additionally, the AKT/mTOR pathway is upstream to the signaling regulating neuronal apoptosis [135, 136].

L-3-n-butylphthalide (L-NBP) is a type of anti-ischemic cranial neuroprotective agent used in CNS diseases, such as ischemic brain injury [137-139]. L-BNP administration protects against VaD through a reduction in hippocampal neuronal apoptosis by suppressing the Bax/Bcl-2 ratio. The potential underlying molecular mechanism involves activation of the PI3K/AKT signaling pathway [140, 141]. However, glial line-derived neurotrophic factor (GDNF)/GDNF family receptor alpha-1 (GFRα1)/receptor tyrosine kinase (Ret) and AKT/ERK1/2 signaling pathways are also implicated in the protective effect of L-BNP against hippocampal neuronal apoptosis in VaD. GDNF/GFRα1/Ret signaling and neuronal viability can be maintained by L-NBP, which activates p-AKT and p-ERK1/2 and decreases the Bax/Bcl-2 ratio and the expression of cleaved caspase-3 [142]. However, recent studies have also found that L-NBP can exert protective effects against cognitive deficits in VaD rats through the inhibition of autophagy (decreased LC-3 and Beclin-1) via mTORC1 pathway up-regulation [143]. This implies that L-NBP is a promising multiple-target drug for the treatment and prevention of VaD. Additionally, the CB2R agonist, WIN, protects against cognitive impairment in CCH-VaD rats via PI3K/AKT signaling [144]. WIN administration can also ameliorate hippocampal neuronal damage after CCH by altering apoptosis signal-regulating kinase 1 (ASK1)-p38 signaling. After the administration of WIN, the expression of phosphorylated ASK1 and p38 is markedly reduced, leading to decreased caspase-9 and caspase-3 expression in the hippocampus of VaD rats [145].

**Figure 3.**
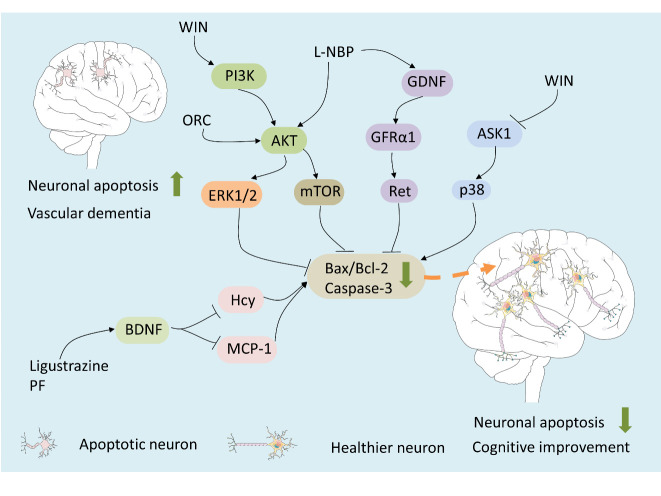
Drugs treat VaD through regulating apoptosis-related pathways. The mechanisms of neuronal apoptosis are of great significance for understanding the pathogenesis of VaD. Similar to inflammation-related pathways, neuronal apoptosis also involved in several pathways. Administration of WIN can inhibit apoptosis through activating the PI3K/AKT pathway and blocking the ASK1/p38 pathway. Besides, the L-BNP can also inhibit apoptosis through GDNF/GFRα1/Ret and AKT/ERK1/2 pathways. The BDNF serves as an anti-apoptotic factor by down-regulating the pro-apoptotic factors MCP-1 and Hcy. Drugs, such as ligustrazine and PF, can suppress neuronal apoptosis by increasing BNDF levels. ORC also could inhibit neuronal apoptosis by up-regulating the AKT/mTOR pathway. The results of the aforementioned drugs will lead to a decrease in the Bax/Bcl-2 ratio and the expression of caspase-3. These effects are likely to improve cognitive deficits in VaD.

Administration of ligustrazine (extracted from *Ligusticum Chuanxiong Hort*.) attenuates VaD via the regulation of BDNF, MCP-1, and Hcy, leading to a low Bax/Bcl-2 ratio and caspase-3 inactivation [126]. These data suggest ligustrazine as a promising neuroprotective drug in VaD therapy. In addition, PF administration suppresses the Bax/Bcl-2 ratio by increasing BDNF levels in the hippocampus of VaD rats [146]. Finally, oxiracetam (ORC) is a nootropic drug that improves cognitive and memory deficits [147]. Studies have found that the administration of ORC markedly mitigates learning and memory deficits and neuronal damage in VaD rats via the inhibition of neuronal apoptosis (decreased Bax/Bcl-2 ratio) and autophagy (decreased LC-3 and Beclin-1) by up-regulating the AKT/mTOR pathway, thereby improving dementia symptoms [148]. These data suggest that ORC may become an effective drug for the treatment of VaD (Fig. 3).

## 5. Restoration of synaptic plasticity

Synaptic plasticity has long been considered as the neurobiological basis of cognition [149]. Synaptic plasticity also plays an essential role in VaD. Synapse damage occurs in the early stages of VaD, and the degree of damage is related to the degree of cognitive impairment. Studies have shown that, in animal models of VaD, synaptic plasticity dysfunction is closely related to cognitive dysfunction [150, 151]. As major factors in the regulation of synaptic plasticity, N-methyl-D-aspartic acid receptor (NMDAR; two subunits, NR2A and NR2B) and calmodulin-dependent protein kinase II (CaMKII) are closely associated with learning and memory functions [152, 153]. In addition, long-term potentiation (LTP) can enhance synaptic transmission, which is closely related to hippocampal learning and memory formation [154]. Studies have found that LTP induction is damaged in the hippocampus of VaD rats and is correlated with reduced CaMKII protein levels [155]

**Table 1:** Drugs treat VaD through multiple targets mentioned in this article.

Drug treatment	Dose	Period	Oxidative stress	Potential molecular mechanism			Synaptic plasticity	Experimental VaD model	Effects on VaD	Ref.
				Central cholinergic system	Neuro-inflammation	Neuronal apoptosis				
TBN	30 mg/kg, i.v.	7 days	4-HNE ↓		Glial activation ↓	Bax/Bcl-2 ↓, caspase-3 ↓		*In vivo* 2VO rats	CBF ↑, learning and memory ↑	[20]
ALA	50 mg/kg, i.p.	28 days	MDA and ROS ↓; GSH ↑	AchE ↓, ChAT and Ach ↑				*In vivo* 2VO rats	Learning and memory ↑	[22]
Betaine	12 mg/kg, i.g.	14 days	MDA and ROS ↓; SOD and GSH ↑				PSD93,95 and MAP2 ↑	*In vivo* 2VO rats	Learning and memory ↑	[23]
BA	10 and 15 mg/kg, i.g.	7 days	MDA ↓; GSH ↑	AchE ↓	TNF-α and IL-1β ↓		BDNF ↑	*In vivo* 2VO rats	CBF ↑, learning and memory ↑	[24]
BCA	2.5 and 5 mg/kg, i.g.	14 days	TBARS ↓	AchE ↓				*In vivo* dyslipidemic rats	Learning and memory ↑	[27]
TWK10	Flexible dose	35 days	MDA and DPPH ↓; CAT, SOD and GSH ↑	AchE ↓				*In vivo* hypertension rats	Learning and memory ↑	[33]
Edaravone	5 mg/kg, i.p.	35 days	MDA and ROS ↓; SOD ↑				PSD93, 95 ↑; CREB and NR2B ↑	*In vivo* 2VO rats	CBF ↑, spatial memory ↑	[37]
Simvastatin	50 mg/kg, p.o.	32 days	MDA ↓; GSH ↑	AchE ↓; Ach ↑	IL-6 ↓; IL-10 ↑			*In vivo* L-methionine rats	Learning and memory ↑	[39]
Melatonin	20 μg/ml, i.g.	28 days	TBARS ↓; GSH, SOD, CAT and TAC ↑	AchE ↓		Bax/Bcl-2 ↓		*In vivo* 2VO rats	Learning and memory ↑	[41, 83]
SLT	50 μg/mL; 16.5 and 33 mg/kg, i.g.	24 h; 28 days	ROS ↓; SOD ↑		Glial activation ↓	Bax/Bcl-2 ↓; caspase-3↓		*In vitro* EAhy926 cells; *In vivo* 2VO rats	No data	[42, 94]
ANF	10 mg/kg, i.p.	5 days	TBARS ↓; GSH ↑	AchE ↓				*In vivo* 4VO rats	Learning and memory ↑	[44]
ICCB	Flexible dose	6 days	MDA ↓; SOD ↑			Bax/Bcl-2 ↓		*In vivo* hyperlipemia rats	Learning and memory ↑	[45]
Resveratrol	Flexible dose	7 days	ROS ↓; SOD ↑	AchE ↓		Bax/Bcl-2 ↓; caspase-3↓	PSD93,95 ↑; CREB and NR2A/B ↑	*In vivo* 2VO rats	Learning and memory ↑	[54, 83, 129, 158]
Chitosan	375 and 700 mg/kg, i.g.	28 days	ROS ↓		Glial activation ↓	Caspase-3 ↓		*In vivo* 2VO rats	Learning and memory ↑	[55]
DM	Flexible dose	37 days	SOD ↑		Glial activation ↓			*In vivo* 2VO rats	Learning and memory ↑	[56]
PNC	5 and 10 mg/kg, i.g.	35 days	MDA ↓; GSH and SOD ↑			Caspase-3 and -9 ↓		No data	Learning and memory ↑	[61]
ST09	3.5 mg/kg, i.p.	42 days	MDA ↓; SOD ↑	AchE ↓				*In vivo* 2VO rats	Learning and memory ↑	[82]
*F. mume*	200 mg/kg, i.g.	21 to 42 days		ChAT ↑	IL-1β and IL-6 ↓; glial activation ↓			*In vivo* 2VO rats	Learning and memory ↑	[93]
WIN	1 mg/kg, i.p.	28 days			IL-1β and TNF-α ↓	Caspase-3 and -3 ↓		*In vivo* 2VO rats	Learning and memory ↑	[98]
PF	Flexible dose	28 days			IL-1β, IL-6 and TNF-α ↓	Bax/Bcl-2 ↓; caspase-3↓		*In vivo* 2VO rats	CBF ↑, learning and memory ↑	[99, 100, 146]
Carnosine	200 and 500 mg/kg, i.p.	37 days	TBARS ↓; GSH ↑	AchE ↓	Glia activation ↓			*In vivo* 2VO rats	Learning and memory ↑	[105]
ReA	40 and 80 mg/kg, i.p.	14 days	ROS ↓		IL-1β, IL-6 and TNF-α ↓	Caspase-3 ↓		*In vivo* 2VO rats	Learning and memory ↑	[122]
L-NBP	15 mg/kg, i.g.	20 days	ROS ↓			Bax/Bcl-2 ↓		*In vitro* HT22 cell; *in vivo* 2VO rats	Learning and memory ↑	[140]
ORC	100 and 200 mg/kg, i.g.	28 days				Bax/Bcl-2 ↓		*In vivo* 2VO rats	Learning and memory ↑	[148]
ET	20 mg/kg, i.g.	7 days	MDA ↓; SOD ↑			Bax/Bcl-2 ↓	CaMKII, NR2B and PSD95 ↑	*In vivo* 2VO rats	Learning and memory ↑	[156]
CBL	2.5 ml/kg, i.p.	28 days				Bax/Bcl-2 ↓	p-CREB and PSD95 ↑	*In vivo* 2VO rats	Learning and memory ↑	[159]

i.v., intravenous; i.p., intraperitoneal; i.g., intragastric; 2VO, 2-vessel occlusion; CBF, cerebral blood flow

Etidronate (ET) administration regulates Ca^2+^ activities, such that Ca^2+^ enters the postsynaptic membrane via NR2B receptor activation and p-CaMKII subsequently improves LTP induction. Moreover, the expression of LTP-related factors, CaMKII, NR2B, and PSD95, is increased by ET administration [156]. In addition, the administration of neuronal gastrin-releasing peptide (GRP) significantly alleviates the cognitive deficits, restores damaged synaptic plasticity, and elevates the expression of the synaptic proteins, synaptophysin (SYP) and CaMKII [155].

The administration of vitamin B6 has been reported to enhance cognitive functions in VaD rats. Additionally, vitamin B6 administration elevates the expression of NR2B, postsynaptic densities (PSDs) protein 95, and CaMKII, which is reduced in the hippocampus of VaD rats. The positive effects of vitamin B6 against VaD are related to the activation of NR2B-dependent PSD50/CaMKII signaling [157]. Additionally, resveratrol, a natural phenolic compound, alleviates cognitive deficits in CCH-induced VaD rats. Administration of resveratrol enhances LTP induction and rescues the loss of dendritic spine and synaptic proteins, possibly via protein kinase A (PKA)/cAMP-responsive element-binding protein (CREB) signaling activation [158]. Cerebrolysin (CBL), a peptide preparation derived from the porcine brain, has beneficial properties similar to those of endogenous neurotrophic factors. Administration of CBL markedly elevates the levels of synaptic proteins, such as PSD95, protein kinase C subunit gamma (PKCγ), and p-CREB, indicating that CBL may alleviate cognitive dysfunction by restoring synaptic plasticity [159]. Additionally, BDNF plays an important role in synaptic plasticity. BDNF can induce LTP and is closely associated with learning and memory processes [160]. Hydroxysafflor yellow A (HYSA), a mixture containing water-soluble chalcones, is a natural pigment of the safflower, *Carthamus tinctorius L*. [161]. HSYA may enhance the levels of BDNF and NR2B, improve LTP at CA3-CA1 synapses in the hippocampus, and alleviate cognitive deficits in VaD rats [162]. The above research results show that pharmacological therapies targeting synaptic plasticity have significant prospects in treating cognitive impairments in VaD.

## Conclusion and Prospects

In summary, since VaD is a neurodegenerative disease characterized by progressive cognitive decline, the ideal drug should be able to prevent or delay the development of VaD. At present, there is not much controversy regarding the fact that VaD is a combined outcome of various pathophysiological changes (such as oxidative stress, central cholinergic system dysfunction, neuroinflammation, neuronal apoptosis, and synaptic plasticity dysfunction) induced by CCH. In this review, we found that many drugs that are beneficial to VaD and the underlying molecular mechanisms target two or more of the aforementioned pathophysiological changes (Table 1). Therefore, drugs aimed at multiple pathophysiological factors may be the future direction in VaD treatment. In addition, we found that natural drugs have been shown to improve cognitive impairments in VaD through a variety of means; their targets and mechanisms were far more complex than those for a single chemical. However, most drugs are still in the basic research stage; thus, it is necessary to attentively await research experiments at the clinical stage in the future.
